# Modulation of Nrf2 and Mitochondrial Function: Pharmacological Implications

**DOI:** 10.3390/ph18111698

**Published:** 2025-11-08

**Authors:** Luciano Saso, Ilker Ates, Ramazan Tunc, Beyza Yilmaz, Marialucia Gallorini, Simone Carradori, Sibel Suzen

**Affiliations:** 1 Department of Physiology and Pharmacology “Vittorio Erspamer”, Sapienza University of Rome, P. le Aldo Moro 5, 00185 Rome, Italy; luciano.saso@uniroma1.it; 2 Department of Pharmaceutical Toxicology, Faculty of Pharmacy, Ankara University, 06560 Ankara, Türkiye; 3 Department of Pharmaceutical Chemistry, Faculty of Pharmacy, Ankara University, 06560 Ankara, Türkiye; eczramazantunc@gmail.com (R.T.); beyza.yilmaz@ankara.edu.tr (B.Y.); sibel2264@gmail.com (S.S.); 4 Department of Pharmacy, “G. d’Annunzio” University of Chieti-Pescara, 66100 Chieti, Italy; marialucia.gallorini@unich.it (M.G.); simone.carradori@unich.it (S.C.)

**Keywords:** Nrf2, oxidative stress, antioxidant, mitochondrial function, ROS

## Abstract

Mammalians are constantly exposed to exogenous and endogenous sources of free radicals that have both favorable and harmful effects on the cellular systems. Oxidative stress (OS) is an imbalance of reactive oxygen species (ROS) and antioxidants in the body that can lead to serious cell damage. It is associated with many diseases such as cancer, Alzheimer’s disease and heart disease. **Background/Objectives**: The Nuclear factor-2 erythroid-related factor-2 (Nrf2) is a transcription factor that controls the cellular oxidation state using antioxidant systems in the body and affects mitochondrial activities. Increased Nrf2 levels serve to protect cells from mitochondrial toxins; however, Nrf2 activity is inhibited in mitochondria-related diseases. In addition, Nrf2 is involved in mitochondrial activities for OS control. **Methods**: As mitochondrial wellbeing and activity is the chief controller for cellular metabolism, Nrf2 is a critical regulator for metabolic pathways. Thus, Nrf2 is the chief organizer of protection against OS in the cells. Nrf2 activator molecules support mitochondrial activity by stimulating mitophagy and helping to battle OS-related permeability transition. **Conclusions**: This review describes the influence of Nrf2 on OS and the way Nrf2 modulates mitochondrial function. Furthermore, we highlight recent studies of Nrf2 regarding its possible role in cell systems as well as pharmacological implications. Furthermore, this review emphasizes the importance of the mitochondria in the development of life-threatening diseases; pharmacological activation of Nrf2 is an important strategy to counter mitochondrial dysfunction

## 1. Introduction

In a healthy cell, the redox state and balance are vital. This balance must be maintained between oxidants, reactive oxygen species, and antioxidants. Oxidative stress occurs when this balance is disrupted by any internal or external cause [1,2]. A balanced redox system ensures cellular homeostasis and a healthy cell cycle. Reactive oxygen species (ROS) consist of both radical species (e.g., superoxide (O_2_^−^) and hydroxyl radical (HO) and non-radical species (e.g., hydrogen peroxide (H_2_O_2_)). Furthermore, reactive nitrogen species (RNS) can also play a role in oxidative stress. Examples include nitric oxide (NO) and peroxynitrite (ONOO^−^) [3,4].

ROS formation can vary significantly depending on their features, the type of cell that produces ROS, and the primary specific biological conditions that cells encounter [5]. Excessive formation of free radicals or excessive exposure alters the balance of ROS and antioxidants. These excess free radicals can damage DNA and cause the degradation of vital elements such as proteins and lipids. ROS is closely associated with the development of neurodegenerative diseases such as Alzheimer’s disease (AD), Parkinson’s disease (PD), and amyotrophic lateral sclerosis (ALS) [6,7,8].

The Nuclear factor erythroid 2-related factor 2 (Nrf2) has been associated with cytoprotective effects in a variety of diseases and encoded by the gene nuclear factor erythroid 2 like 2 (NFE2L2) that belongs to the Cap’n’Collar (CNC) subfamily of basic leucine zipper (bZip) transcription factors, which comprises nuclear factor erythroid-derived 2 (NFE2) and Nrf1, Nrf2, and Nrf3. Nrf2 is responsible for regulating a wide panel of antioxidant enzymes involved in the detoxification and elimination of oxidative stress. It is involved in the prevention of the inflammation mechanism [9]. Nrf2 targets gene encode proteins responsible for the repair and removal of vital elements involved in tasks such as the activity of antioxidant enzymes and the detoxification of ROS [10].

During OS, Nrf2 prevents KEAP1-associated degradation, enters the cell nucleus, and binds to antioxidant response elements (AREs) in the promoter regions of genes [11,12]. Recent research has presented a link between the activities of mitochondria and reactions to oxidant elements. Animal studies have also identified a close link between mitochondrial dysfunction [13], increased oxidative damage [14], and decreased oxidant levels in vital organs such as the brain [15,16].

Mitochondria are organelles that act as energy-producing power plants for the cell, continuously producing energy and acting as bio-synthetic and bioenergy factories. Mitochondria have a major role in the healthy and normal functioning of cells. Different pathways take place in moderating mitochondrial activity, with the help of several proteins in specific mechanisms allowing interaction between mitochondrial metabolism and oxidoreductive homeostasis. The maintenance of mitochondrial homeostasis is linked with the regulation of many functions of the mitochondria. These functions are related to several other crucial physiological processes like programmed cell death, apoptosis, autophagy, metabolism, calcium flux, and immunity. Mitochondria can inhibit or prevent degenerative processes in tissues, significantly reducing the rate of onset of the disease [17,18]. Therefore, mitochondrial dysfunctions might affect cellular bioenergetics, cellular function, and cellular homeostasis, making mitochondria a key player in health and disease [19]. 

The undisputed role of Nrf2 in mitochondrial biogenesis (MB) has been established by many studies, including experiments in animals with Nrf2 deficiency [20]. Under oxidative and electrophilic stress conditions, Nrf2 degradation is inevitable as a result of the chemical modification of KEAP1, which acts as a protector against electrophiles and oxidants. Nrf2 degradation will weaken the antioxidant defense system and will also negatively affect mitochondrial functions and cellular mechanisms.

This review addresses Nrf2–mitochondria interactions and their pharmacological outcomes. From this perspective, Nrf2-modulating molecules and the conditions responsible for this effect are of great importance. The functions of Nrf2 in regulating mitochondrial health and pharmacological responses in changing redox environments are being investigated by many researchers. The general consensus is that mitochondrial dysfunction is involved in the pathology of many diseases [21]. The development of new therapeutic strategies targeting mitochondrial dysfunction has been adopted as a new strategy. In this context, Nrf2, which influences various cytoprotective systems, may be a promising target for countering mitochondrial dysfunction and its consequences. Therefore, exogenous Nrf2-modulating molecules and their pharmacological activities hold therapeutic potential.

## 2. Mitochondrial Function and Cellular Bioenergetics

Mitochondria are universal organelles with very important cellular functions, located in the cell structure of most eukaryotes. They play a role in many vital functions of the cell cycle and have a major role in energy creation. In addition to all these vital functions, mitochondria are known to have roles in many biological events such as apoptosis, homeostasis, and synthesis of heme and iron–sulfur clusters [22]. Oxidative phosphorylation (OxPhos) is known as the characteristic reaction of mitochondria that produces ATP using energy obtained from nutrient oxidation. While mitochondria undertake all these vital tasks, Nrf2 helps as an important regulator of mitochondria’s numerous functions in the cell [23].

Mitochondria produce almost 90% of cellular ROS [24] and almost 90% of the cellular energy by ATP through the tricarboxylic acid cycle (TCA) and the electron transport chain (ETC). Mitochondria are responsible for crucial tasks, containing production of energy, signal transduction, cellular homeostasis, and apoptosis [25,26]. Mitochondrial diseases are considered as orphan illnesses that are very well described genetically, and they are not easy to cure. Animal studies revealed that some mitochondrial diseases prolonged survival through the exposure to chronic hypoxia [27]. Mitochondrial ROS are largely created at the ETC by the procedure of OxPhos and through reduction of oxygen to H_2_O [28]. The major origin of mitochondria-based superoxide are Complex I and III. Mitochondrial Complex I (NADH:ubiquinone oxidoreductase) is responsible for the mitochondrial respiratory chain that provides electron transport and the production of a proton rise through the mitochondrial inner membrane to ATP generation [29,30]. Mitochondrial Complex II is a significant actor in the aging progression as it transforms succinate to fumarate and shows a crucial role in both the TCA and the ETC [31]. Mitochondrial complex III produces superoxide through the ubiquinone (Q)-cycle [32]. This cycle transfers two electrons to ubiquinone from complex I and complex II and results in the reduction of ubiquinone to ubiquinol (QH2) [33,34]. The generation of ROS in the ETC is reliant on the presence of electrons from the electron transport cascade by the formation of ROS. This procedure could be prompted by the accomplishment of mitochondrial complexes I and II as donors of electrons [35].

The mitochondrial thioredoxin (Trx)/peroxiredoxin system contains several essential structures. The system operates primarily in the presence of NADPH, thioredoxin reductase 2 (TrxR2), thioredoxin 2, and peroxiredoxins 3 and 5 (Prx3 and Prx5). Furthermore, the function of these particles is crucial for regulating cellular redox homeostasis through the efficient catabolism of peroxides [36]. Trx and GSH-related systems in the body play a crucial role in maintaining redox balance, particularly in tissues that are highly energy-dependent and more prone to oxidative stress, such as the brain. By reversibly regulating thiol modifications in cells, Trx and GSH systems also control redox signaling, which plays a role in various biological processes in the central nervous system. Mitochondria are equipped with antioxidant defense elements and contain high concentrations of glutathione (GSH). This system has a complex structure and is still not fully characterized. Many elements play critical roles in this system. In addition to Prx3, Prx5, Trx2, and TrxR2, Mn superoxide dismutase (SOD) (MnSOD), glutaredoxin 2a (Grx2a), the long form of GSH peroxidase 4 (L-GPx4), and uncoupling protein (UCP) 2 (UCP-2) also play important roles [37].

**Scheme 1:**
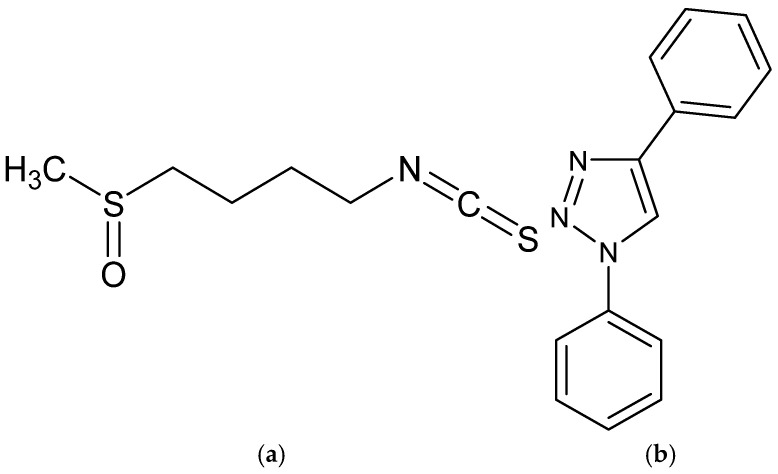
Sulforaphane (**a**); 1,4-diphenyl-1,2,3-triazole (**b**).

During homeostasis, Nrf2 has important support functions. These include vital activities such as changing the membrane potential of mitochondria, oxidation of fatty acids, and ensuring the availability of substrates for respiration. It was found that compounds that activate Nrf2, particularly naturally occurring sulforaphane (SFN) (Scheme 1), prevent the opening of the mitochondrial permeability transition pore. SFN, an Nrf2 activator, has been found to increase Prdx6, catalase, and GSTπ expression in a dose-dependent manner and to enhance Nrf2/DNA binding by facilitating Nrf2 translocalization in the nucleus. It was found that SFN can mitigate Prdx6 loss due to ARE/Nrf2 dysregulation, suggesting that this strategy may offer hope in the treatment of aging-related diseases [38]. SFN can directly inhibit NF-κB activity, leading to the downregulation of pro-inflammatory genes and inflammasomes. It also regulates glucose levels and insulin resistance and has lipid-lowering effects [39]. Some synthetic molecules (e.g., 1,4-diphenyl-1,2,3-triazole) promote total mitochondrial homeostasis by motivating mitophagy. These entities highlight Nrf2 as an important actor in linking the structural and functional integrity of mitochondria [40].

Aging and similar phenomena cause mitochondrial respiration and cytochrome c functions to weaken and regress, and fail to show sufficient activity. However, mitochondrial ROS production for the oxygen consumed and mitochondrial indicators of OS were raised in the skeletal muscle, which also displayed lesser expression of Mfn1 (mitofusin 1) and Mfn2 (mitofusin 2) mRNAs [23]. Mitochondrial functions are mostly affected by Nrf2 via moderating the substrates (NADH and FADH2) for mitochondrial respiration. These substrates are also crucial for antioxidant enzymes to act. Nrf2 has an important role in the proper functioning of the relationship between OxPhos and the redox state of the cell. It also works to regulate cell activities through Nrf2/KEAP1 action. From this perspective, stimulation of the Nrf2/KEAP1 pathway can be considered as a new therapeutic approach for mitochondrial dysfunction and diseases associated with OS.

In recent years, studies in the field of cancer have shown that OxPhos has an effect on tumor formation [41,42]. It has been observed that OxPhos slows down or prevents the development of cancer cells as a result of suppression by different means [43]. OxPhos ensures the healthy development of cells and controls apoptosis and cellular proliferation rate when necessary [44]. The difference in energy production mode in a normal and carcinogenic cell, which develops depending on the mitochondria, is a very important biomarker for the analysis of tumor development and stages [45].

The organism has many protective mechanisms to prevent damage to the mitochondria. However, despite this, mitochondrial dysfunction can continue and eventually trigger cell death. Mitochondrial dysfunction primarily affects OxPhos function. In addition, it is associated with many diseases that threaten human health, such as diabetes, cancer, aging, and various neurodegenerative diseases. Mitochondrial bioenergetics and metabolism are very important for the activities of cells and the healthy functioning of the cell. A healthy life is directly related to the homeostasis associated with mitochondria, mitochondria-associated membranes (MAMs), and their activities in inflammation, and cell death emphasizes their importance. Disorder of mitochondrial bioenergetics and dynamics, ER–mitochondria crosstalk, and increased superoxide contribute to various pathologies in patients [46]. Understanding these complex mitochondrial tasks and elucidating their mechanisms can be used as a new approach to the treatment of mitochondria-related diseases [47].

Mitochondria are critically important in cancer development because they participate in and control many cellular processes. Genetic and epigenetic changes in mitochondrial and nuclear DNA are crucial. These changes disrupt oxidative phosphorylation, the tricarboxylic acid cycle, and redox homeostasis. This is known to trigger tumor development. When examining cancer types, mitochondrial dysfunction can differ among individuals. This impacts treatment responses. While targeting mitochondrial function is a promising strategy for cancer therapy, it is still under investigation [48]. Recent studies have shown that Nrf2-dependent processes in metabolism and homeostasis affect mitochondrial activity. Nrf2 is known to be activated by abnormal accumulation of the TCA. Therefore, in the absence of Nrf2, ATP production and oxygen consumption in cells of some tumor types have been found to be reduced [49]. We know that Nrf2 plays a major role in mitochondrial functions, particularly in essential processes such as biogenesis, ATP production, and mitophagy. NRF1, on the other hand, is a transcription factor for many genes involved in mitochondrial biogenesis during exposure to oxygen. Nrf2 also contributes by promoting the transcription of NRF1 and TFAM. Furthermore, NRF1 increases TFAM expression through the participation of peroxisome proliferator-activated receptor coactivator-1 (PGC-1). Thus, TFAM and PGC-1 play a role in mitochondrial biogenesis [50]. Numerous molecules that inhibit tumor formation or growth, affect mitochondria, and interact with the Nrf2/Keap1 pathway are currently being developed. The Nrf2/Keap1 pathway has extensive interactions with mitochondria, and in cancer cells, glycolysis/OXPHOS is closely linked to Bach1, in addition to Nrf2 and p21, and Keap1 is closely linked to mitochondrial biogenesis/mitophagy in metastatic CRC tumors, attempting to identify new drug targets [51]. On the other hand, the physiological environment of tumor cells is quite different from that of healthy cells. Therefore, to adapt to adverse conditions such as hypoxia, tumor cells must reprogram metabolic pathways to maintain vital functions such as energy, biosynthesis, and redox processes. The “Warburg effect” is one of the most common modes of reprogramming. Even when oxygen is sufficient, tumor cells primarily target glycolysis for ATP production and switch their metabolic modes when stress conditions differ. As studies on cancer treatments progress, metabolic reprogramming is being evaluated as a new strategy for metabolic changes in cancer cells [52].

## 3. Regulatory Interactions Between Nrf2 and Mitochondria

In apoptosis, biosynthesis and cellular bioenergetics formation, which are among the most important activities of the organism, and regulatory cross-effects between Nrf2 and mitochondria are of critical importance. Mitochondria are structured by fusion in response to changes in metabolic events, ensuring a healthy homeostasis. During these actions, the damaged mitochondria are destroyed by mitophagy. This is an important protection mechanism [53]. As a protective mechanism, PERK (Protein Kinase RNA-Like ER Kinase)-mediated Nrf2 activation following ER stress protects mitochondria by preserving their metabolism, dynamics, and quality control. Although the mechanism has not been elucidated in detail, we know that activation of PERK promotes translation inhibition and cell cycle arrest after accumulation of unfolded proteins in the endoplasmic reticulum (ER). The transcription factor Nrf2 is a PERK substrate. In unstressed cells, Nrf2 is retained in the cytoplasm through association with KEAP1 [54,55].

PERK functions as an endoplasmic reticulum (ER) stress sensor that responds to the accumulation of misfolded proteins. PERK activation also activates signaling pathways that maintain and support redox homeostasis. There is a PERK–mitochondria axis characterized by PERK sensing stress in the ER and mitochondria and it prevents apoptosis. During ER stress, PERK activation can prevent apoptotic fragmentation of mitochondria, increase cellular ATP production capacity, and strengthen mitochondria by enhancing mitochondrial biogenesis and mitophagy [56].

In order for mitochondria to maintain their activities, a balanced relationship between calcium and ROS homeostasis, and mitochondrial turnover (biogenesis/mitophagy) and mitochondrial fission/fusion is required [57,58]. Nrf2 is a critical regulator of MB together with the transcription factor Nrf2 (nuclear respiratory factor 1) and transcription coactivators PGC-1α (peroxisome proliferator-activated receptor-γ coactivator) and PGC-1β. In addition to TFAM (mitochondrial transcription factor A), which regulates the transcription of respiratory chain genes expressed in mtDNA, they are also involved in the transcription of nuclear gene encoding mitochondrial proteins [59,60].

The level of ROS generated directly affects the activity of Nrf2, which is a nuclear factor sensitive to OS. This interaction can disrupt the functioning of many physiological processes such as glucose and lipid homeostasis [58]. In other words, when the gene encoding Nrf2 is not functioning under certain conditions, the amount of ATP required for the cell decreases [61]. In the event of exposure to oxidants, Nrf2 binds to the UCP3 promoter and then to the ARE. Therefore, cell survival may be affected by UCP3-mediated proton leak in response to ROS exposure [62]. Among the functions of Nrf2 is the regulation of the NRF2 gene, which has an ARE. NRF1 expression and MB are stimulated in an Nrf2-dependent manner [63]. Nrf2 transcriptional activity has an impact on many activities of mitochondria as well as homeostasis [64]. These actions include MB and bioenergetics [65,66], respiration [67], fatty acid oxidation, ATP production [68,69], membrane potential and redox homeostasis [70,71], and protection against OS [72,73,74].

MB is a defense system developed against the decrease in ATP synthesis in the cell by providing the production of new healthy mitochondria in cells and the repair of damaged ones. mtDNA transcription and mtDNA replication are responsible for the formation of new mitochondria required for this process [75,76,77]. The proteins required for the regulation of mtDNA are encoded and regulated by both nuclear and mitochondrial genes [78,79]. When the mechanism of MB is examined, it is seen that many factors play a role in this process. It is a very complex process that requires tight communication between mitochondrial and nuclear transcription factors. There are important indicators of MB. Some of these can be listed as mtDNA/nDNA ratio and PGC-1α, TFAM, Nrf1, and mitochondrial transcription factor B1 (TFB1M). These are responsible for the expression of gene encoding mitochondrial regulatory proteins. In addition to this task, the synthesis of nucleotides and phospholipids also plays an important role in MB.

**Figure 1:**
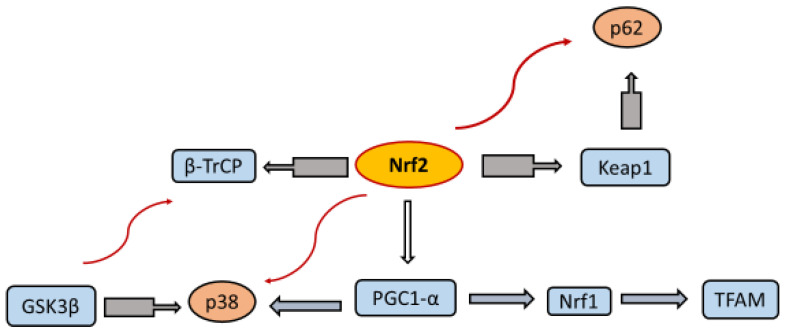
Nrf2 is crucial for maintaining cellular and mitochondrial homeostasis. KEAP1 and β-TrCP are Nrf2 ubiquitination-facilitating regulator proteins and have the potential to increase Nrf2 degradation. The mitophagy-linked regulatory circle (p62 and Nrf2) and the MB-linked regulatory circle (PGC1-α, p38, GSK3β, and Nrf2) are both existing in the basic scheme of mitophagy and MB pathways mediated by p62 and PGC1-α, respectively, upon Nrf2 activation. Arrows indicate stimulation, including catalysis; blunt-ended arrows indicate inhibition [53].

PGC-1α, a main actor of MB, affects the downstream nuclear respiration elements Nrf1 and Nrf2 and then activates TFAM. Deletion of the *N*-terminal fragment in Nrf1 abolishes the PGC-1α effect on mitochondrial biogenesis. Signaling for MB is activated by PGC-1α, resulting in the upregulation of proteins encoded by both nuclear and mitochondrial genomes [80,81]. Animal experiments have shown that the protein encoded by the Nrf2 target gene stimulates MB. MB declines with age. This might be due to modifications in mitochondrial fission and fusion progressions and the inhibition of mitophagy that causes the elimination of dysfunctional mitochondria. The occurrence of MB and its relationship with mitophagy are briefly shown in Figure 1.

Unhealthy or malfunctional mitochondria are powerless in entering the mitochondrial network and are therefore designated for degradation via mitophagy. Mitochondria are wrapped into double membrane during mitophagy. This membrane attaches to lysosomes, eventually causing the degradation of their internal substances. The control and involvement of mitophagy particularly in neurodegenerative disorders are subjects of big attention with numerous continuing studies but various unanswered problems persist [82,83,84].

Mitochondria have developed essential mechanisms to guard their DNA from harmful substances and physical injuries like DNA restoration activities. Nevertheless, there are mitochondria-related actions like rapid mtDNA income, fission, fusion, and mitophagy. Still, mtDNA mutations could be plentiful in the somatic tissue, affecting OxPhos, as a result of the ROS produced in ATP creation [85]. Deletions in the mitochondrial genome are established in numerous genetic disorders [86] and cancer [87], as well as aging and neurodegenerative diseases [88]. However, compared to the mechanisms controlling mtDNA replication, mtDNA repair pathways are not much described. On the other hand, it has been determined that some proteins responsible for activities in these pathways remain within the mitochondria [89,90].

**Figure 2:**
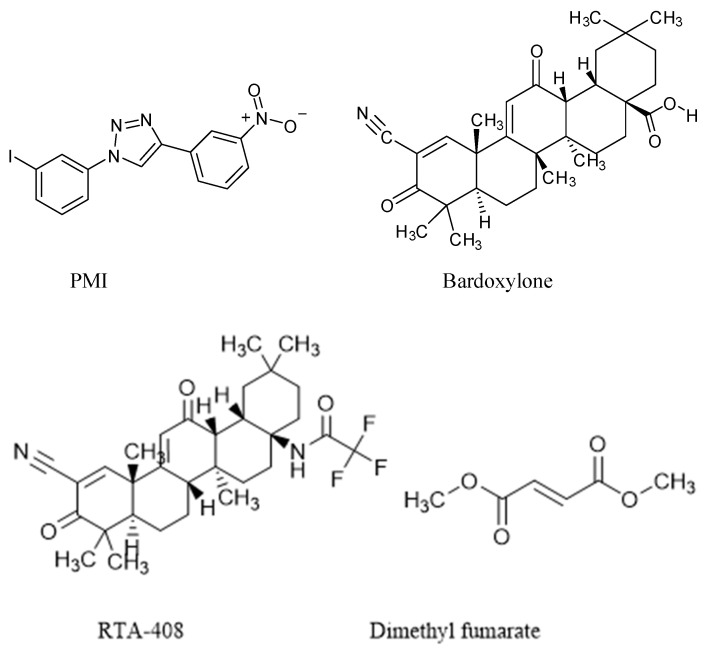
The structures of PMI, bardoxylone, RTA-408, and dimethyl fumarate.

There are some compounds like PMI (P62-mediated mitophagy inducer) [91,92], bardoxylone [93], RTA-408 [94], and dimethyl fumarate (DMF) [95] that act as Nrf2 activators and help to develop mitochondrial function. PMI leads mitochondria to perform quality control without cooperating with the bioenergetic capability of the whole network [91,92]. Oleanolic acid derivatives of semi-synthetic compounds bardoxolone and bardoxolone methyl trigger the Nrf2 pathway and prevent the formation of NF-κB [96]. They prevent the inflammation in neurodegeneration that triggers the Nrf2 signaling pathway and controls signaling pathways of NF-κB and Nrf2 [97]. DMF is also a recognized activator of Nrf2. Dissociation of these proteins and the resulting stimulation of Nrf2 are responsible for the mechanism [98,99]. The structures of PMI, bardoxylone, RTA-408, and DMF are demonstrated in Figure 2.

Common Nrf2 activators and their ameliorating effects on mitochondrial dysfunctions that cause disorders are demonstrated in Table 1.

In addition to providing energy through OxPhos in the cell, mitochondria are involved in many bio-synthetic activities. Although the mechanisms of most of these activities are not fully known, they are the subject of many studies [106,107].

Nrf2 can directly or indirectly affect many antioxidant systems and increase their effects [24,25]. The most important of these antioxidant systems are glutathione (GSH) synthesis [26], GSH peroxidases [27], GSH reductase [28], Peroxiredoxin 3 [29], and TRXR2 [30]. Furthermore, Nrf2 plays an important role in MB by affecting the expression of critical transcription factors such as peroxisome proliferator-activated receptor gamma [31]. These interactions increase the resistance of mitochondria to increased oxidative stress. These results also support the thesis that mitochondrial damage may occur in the event of Nrf2 deficiency. Despite all these findings, the relationship and mechanism of mitochondrial oxidative stress and damage through Nrf2 activation have not been fully elucidated. The most accepted possibility is that increased mitochondrial ROS production produces hydrogen peroxide, which passes from the mitochondria to the cytosol and activates Nrf2 directly or indirectly. The second possibility is that redox changes within the mitochondria activate Nrf2 by sending secondary signals to the cytosol [32].

Mitophagy primarily affects the mitochondria that have been depolarized during the apoptosis process. It results in the formation of an isolation membrane surrounding the cell organelles and cytoplasm, which are sent to lysosomes for digestion and recycling. In addition, mitophagy directs damaged mitochondria to autophagy [33,34]. As a result, autophagy is of primary importance for the health and survival of cells and also allows for the proper functioning of homeostasis. The disruption of the perfect relationship between mitochondria and autophagy can lead to the formation of metabolic and autophagic diseases [35].

### Lipid Peroxidation and Ferroptosis

Decreased detoxification of lipid peroxides through the enzyme activities of glutathione peroxidase 4 (GPX4) [108] or the lack of this ability appears to be the primary cause of ferroptosis. Furthermore, these findings demonstrated that ferroptosis was primarily caused by the peroxidation of PUFAs. When lipoxygenases oxidize polyunsaturated fatty acids (PUFAs), peroxides build up and may help produce lipid peroxide products from their breakdown. Conversely, oleic acid, a monounsaturated fatty acid (MUFA), exhibited an opposing effect of inhibition [109], likely by neutralization that protected ferroptosis, whereas PUFAs, such as linoleic and arachidonic acids, promoted cells to RSL3-induced ferroptosis.

According to latest research by Doll et al. [110], ferroptosis is caused by acyl-CoA synthetase long-chain family member 4 (ACSL4), which builds up oxidized cellular membrane phospholipids. The deadly lipid species that ACSL4 produces oxidized phosphatidylethanolamines (PEs) and were later found to promote ferroptosis by Kagan et al. [111]. Using CRISPR technology, Doll et al. [110] showed that ferroptosis and the generation of lipid peroxides were prevented in cells by knocking down the ACSL4 gene. Transgenic overexpression of ACSL4 in cells where GPX4 functionality was specifically suppressed to avoid a confusing effect reversed this finding. Furthermore, by blocking ACSL4 with thiazolidinedione in a conditional deletion model of GPX4, this evidence was confirmed in vivo. Additionally, despite its complexity, the identification of PE as the oxidized species responsible for ferroptosis was compelling. By analyzing extracts of RSL-3-sensitive cultivated cells using genetic, bioinformatics, and LC-MS/MS techniques, oxidized phospholipids generated during ferroptosis were found [112]. Only a single group of phosphatidylethanolamine (PE) phospholipids has the lipid species that are specific to the development of ferroptosis, rigorously identified both in vitro and in vivo in GPX4 models. It has been demonstrated that PEs containing the fatty acyls arachidonoyl (AA) and adrenoyl (AdA), which are stimulated by ACSL4, are lethal signaling of ferroptosis, especially in mitochondria. AA or AdA esterification into phosphatidylethanolamines (PEs) was reduced by gene deletion and pharmacological inhibition of ACSL4. GPX4 inhibits ferroptosis by reducing lipid hydroperoxides. It has been demonstrated that the antioxidants vitamin E (α-tocopherol) and α-tocotrienol control ferroptosis through LOX suppression, whereas the antioxidant enzyme GPX4 negates lipid peroxides [111]. In spite of scavenging hydroxyl group radicals, vitamin E also competes at the substrate site of attachment to inhibit LOX. The mechanism whereby vitamin E suppresses LOX activity through competing with the substrate at the enzyme’s binding site was outlined using electron spin resonance (ESR) spectroscopy and LC-MS. Despite their inability to produce tocopheroxyl radicals, esterified vitamin E analogs, such as α-tocopherol succinate (TS) or α-tocopherol phosphate (TP), inhibited LOX activity by vying for the PUFA substrate binding site. Ferroptotic death has been demonstrated to be prevented by vitamin E in vitro [108,113,114] and in vivo in Gpx4^−/−^ knockout mice [115,116]. Various organs and conditions linked to ferroptosis are highlighted by tissue-specific expression of 15-LOX and perhaps vitamin E status. Ebselen, Coenzyme Q, Liproxstatin-1, and Ferostatin-1 are other ferroptosis inhibitors. Additionally, various cell types have varying levels of sensitivity and susceptibility to ferroptosis inducers [117]. Certain lymphoma cells depend on external supplies of cysteine and cystine due to defects in sulfur transfer mechanisms. For instance, co-culturing Nb2 lymphoma cells with cysteine-secreting fibroblasts or supplementing them with 2-mercaptoethanol prevented sulfasalazine-induced ferroptosis. Furthermore, in reaction to sorafenib-induced ferroptosis, hepatoma carcinoma cells showed higher concentrations of HO-1, FTH1, and NAD(P)H Quinone Dehydrogenase 1 (NQO) mRNA [118], presumably to lessen the impact of ROS. Essentially, in pathological conditions like Huntington’s disease, acute kidney disease, B cell lymphoma, periventricular leukomalacia, and cancers, GPX4 inhibition, GSH decline, and raised lipoxygenase activity promote PUFA accumulation and the formation of fatty acid radicals, which include MDA and 4-hydroxynonenal, that lead to ferroptosis and death of cells [119].

## 4. ROS Induces Damage to the Mitochondrial Respiratory Chain

To synthesize ADP, which is the primary energy source for cells, a reaction is created by dephosphorylating an ATP. In order for this process to occur and for new ATP to be synthesized, glycolysis with helper molecules is required [120,121,122,123,124].

The generation of ROS in the cellular environment is thought to be caused by mitochondrial malfunction and enrichment of mitochondria with different redox enzymes [125,126,127]. As a byproduct of the respiratory chain, superoxide (O_2_^−^) is created from molecular oxygen in the mitochondria or NADPH oxidase. SOD can convert superoxide to H_2_O_2_. Further production of hydroxyl radicals and hydroxyl anions is facilitated by hydrogen peroxide [128].

**Figure 3:**
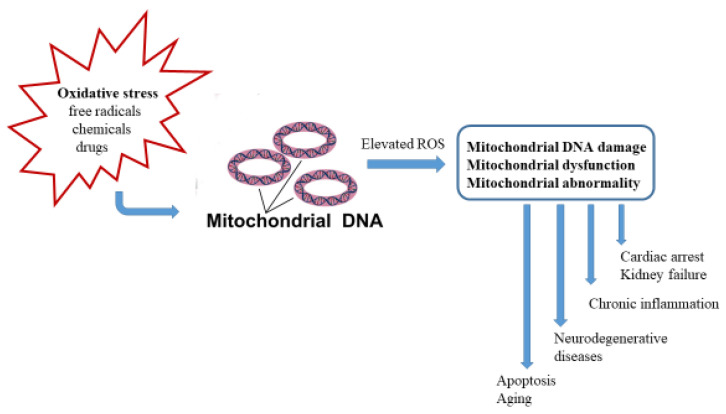
Mitochondrial involvement in oxidative stress and diseases.

Vital molecules such as proteins, lipids, and DNA serve as oxidative damage biomarkers of aging and many neurological diseases. In addition, ROS are known to cause protein carbonyls, lipid peroxidation to malondialdehyde (MDA), and guanine oxidation to 8-oxo-deoxyguanosine in DNA [129]. ROS production mainly occurs in the inner membrane of mitochondria [130]. There are also some known molecules such as superoxide dismutase (SOD) and GPX in the inner membrane. SOD catalyzes the dismutation of superoxide radicals (O_2_^−^) to molecular oxygen and hydrogen peroxide, and GPX catalyzes the reduction of hydrogen peroxide to water and oxygen [131]. The outer membrane of mitochondria is more porous and absorbent to lower molecular weight elements and ions. Molecules targeting mitochondria are believed to be more selective and active to address diseases associated with OS [132,133,134,135]. A schematic representation of ROS-induced mitochondrial dysfunction and involvement in a number of diseases is shown in (Figure 3).

When the free radical production and antioxidant detoxification mechanism in the organism functions regularly, mitochondria are not in danger. If ROS production escalates, this causes the incapacitation of main elements of the respiratory chain and enzymes of the Krebs cycle [136]. Mitochondrial ETC is one of the main sources of ROS production by aerobic respiration in the majority of cells. Wong et al. [137] demonstrate that mitochondria account for 45% of ROS in C2C12 myoblasts and NADPH oxidase account for 40%. Four multi-subunit protein complexes (CI, CII, CIII, and CIV) make up the respiratory chain, also known as the mitochondrial ETC that is encased in the mitochondrial inner membrane. The ATP-generating OxPhos in the mitochondrial system is made up of the F1F0-ATP synthase and the ETC [138]. All OxPhos complexes, with the exception of CII, are made up of subunits that are encoded by mitochondrial (mtDNA) and nuclear (nDNA) DNA [139]. The two forms of ROS, which are superoxide (O_2_^−^) and hydrogen peroxide H_2_O_2_, are created during the mitochondrial respiratory chain [140]. Nevertheless, many questions regarding the mechanisms of O_2_^−^ generation, particularly in physiological conditions, remain unanswered despite extensive biochemical and biophysical investigations on electron and proton transport through the respiratory chain [137,141,142,143,144]. The injury of the mitochondrial respiratory chain can create a significant rise in intracellular ROS. Due to the excess ROS level, it will worsen mitochondrial damage and lower the antioxidant capacity of cells [145,146].

It is now commonly acknowledged that, in addition to mitochondria, the NADPH oxidase system is one of the main sources of ROS in cells and a crucial contributor to intracellular ROS homeostasis [147]. The NADPH oxidase was first identified as the enzyme that causes the oxidative burst that leukocytes use to destroy bacteria [148], but it is now thought to be involved in nearly every tissue kind [149]. This enzyme has several isoforms (NOX1–NOX5 and DUOX1 and 2) and its expression of these subtypes differs depending on the tissue type [149]. Although NOX4 also appears to be involved [150] in brain damage, NOX2 is the primary variant of NADPH oxidase in brain tissues such glia and neurons [151,152]. Brain abnormalities have been associated with NOX2 and NOX4 stimulation, suggesting that ROS production via NADPH oxidase plays a role in both acute and chronic neurological conditions [153,154]. Numerous lines of proof clearly imply that Nrf2 activation is regulated by NADPH oxidase; in fact, NOX4 in cardiomyocytes and pulmonary epithelial cells has been demonstrated to do so [155,156]. It is far less clear if the opposite is true, that is, whether the KEAP1–Nrf2 route controls the expression and activity of NADPH oxidase. The best genetic system of study for addressing the impact of Nrf2 on NADPH oxidase expression and testing the theory that Nrf2 exertion and NADPH oxidase expression reciprocally cooperate to create a negative feedback regulatory loop is animals and cells with graded expression of Nrf2, such as Nrf2-knockout (Nrf2-KO), wild-type (WT), and KEAP1-knockdown (KEAP1-KD) [157]. Since the KEAP1–Nrf2 pathway is being targeted more and more for the prevention and cure of human disease, and because numerous clinical trials have been completed or are in progress, this information is crucial [158]. Furthermore, the Nrf2 antioxidant pathway is activated by BG12, which is an oral bioavailable formulation of DMF that is approved for the medical management of multiple sclerosis [159].

## 5. Nrf2 Modulation

Transcriptional factor Nrf2 is connected with the vital protection of the cells against the expression of cytoprotective genes by oxidants [160,161]. Nrf2 arranges cellular defense progress against oxidant molecules through controlling the expression of more than 500 genes. This mechanism is linked to antioxidant compounds, detoxification procedures, or the presence of metabolic enzymes. The two degrons that are specifically associated with the Neh2 domain are made by the KEAP1 [162,163,164].

Nrf2, a member of the Cap’n’Collar family of essential leucine zipper transcription factors, binds to AREs to activate the transcription of phase II detoxifying antioxidant enzymes such as heme oxygenase-1 (HO-1), glutamate-cysteine ligase (GCL), and NAD(P)H:quinone oxidoreductase-1 (NQO1).

**Figure 4:**
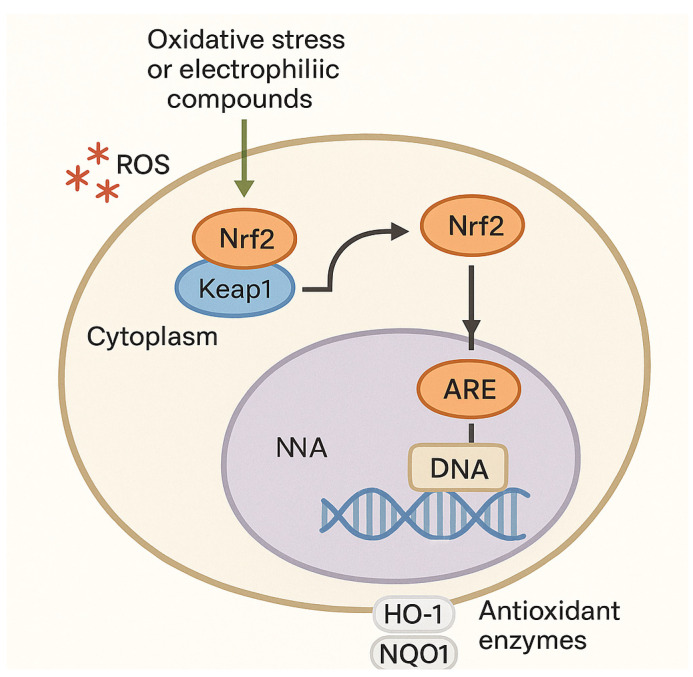
Proposed modulation of Nrf2.

Nrf2 facilitates cellular adaptation to redox stress that cells encounter. When signals arrive, Nrf2 enters the cell nucleus from the cytosol and binds to the ARE. This activates the expression of antioxidant genes and cytoprotection against damage caused by oxidative stress. It induces a decrease in sensitivity to damage. This Nrf2 activity is an important target in the treatment of diseases associated with oxidative stress such as inflammation, cancer, fibrosis, and obesity (Figure 4). In addition, Nrf2 modulation plays an important role in the treatment of diseases such as liver inflammation, fibrosis, and cancer due to target gene induction. An important path can be made in the treatment of many related diseases by inhibiting oxidative stress and NLRP3 inflammasome activation [165].

**Figure 5:**
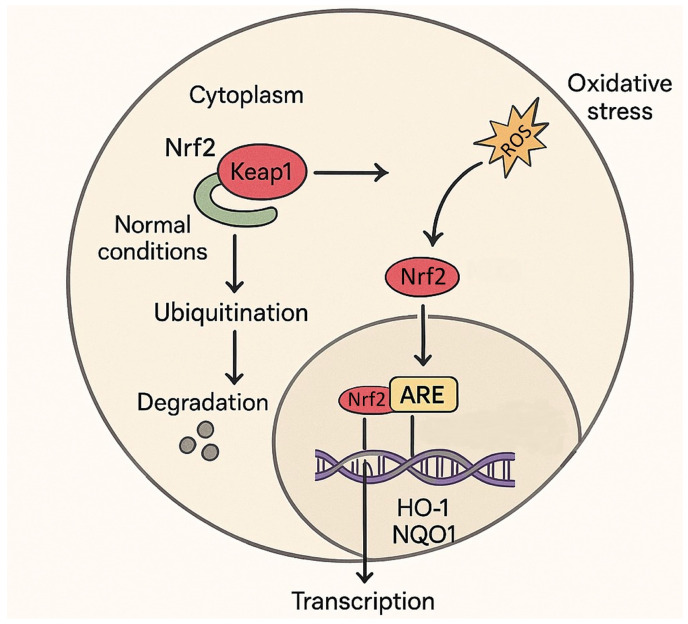
Schematic representation of the Nrf2-KEAP1-ARE signaling pathway.

Animal experiments have shown that Nrf2 activation can improve energy expenditure and prevent weight gain in mice [166]. In addition, it has been shown that Nrf2/HO-1 pathway activation can prevent cognitive impairment, inflammatory events, and Aβ and tau accumulation in the brain [167]. Nrf2 activity also has the capacity to prevent obesity-related vascular oxidative damage and increased ROS production [168,169]. However, it should not be forgotten that aging is closely related to dysfunction in the Nrf2/ARE signaling pathway (Figure 5). This disorder triggers pro-oxidative activity and plays a major role in neurodegenerative diseases and aging [170].

During normal cell functioning, Nrf2 is continuously degraded by the proteasome via the formation of the KEAP1 complex. With OS exposure, KEAP1 is inactivated. This is followed by phosphorylation and accumulation of Nrf2 in the cell nucleus. Here, it binds to ARE regions and activates many genes, primarily antioxidants and detoxifying enzymes.

Nrf2 is responsible for controlling biosynthesis of GSH, GSH-regenerating enzyme, and glutathione reductase (GR), as well as antioxidant protection genes [171]. Furthermore, activation of Nrf2 and stimulation of mitochondrial antioxidant enzymes are connected, emphasizing the role of Nrf2 in maintaining mitochondrial redox homeostasis [172]. Nrf2 target genes have positive influence on mitochondria, using diverse actions including maintaining the defense against OS, mitophagy, and mitochondriogenesis [173,174].

The most significant intracellular antioxidant mechanism is the Nrf2/ARE signaling route [175,176]. Moreover, the Nrf2/ARE pathway can stimulate the production of several downstream antioxidant enzymes, such as SOD 1 and heme oxygenase 1, which can lower the effects of OS and prevent cell injuries [177]. But in end-stage diabetes, the endogenous antioxidant system’s damage inevitably results in Nrf2 attenuation.

Nicotinamide adenine dinucleotide (NAD+) is required for the deacetylase enzymes that make up the family of mammalian mute information regulator two proteins, or Sirtuins. SIRT1, a class I histone deacetylase through deacetylation in an NAD+-dependent manner, can also be a driver of the antioxidant response. Primarily found in the nucleus, SIRT1 can interact and deacetylate Forkheadbox protein O3 (FOXOa3) and PGC1α as well as histones [178,179]. Enhanced transcriptional action and promotion of biogenesis in mitochondria and antioxidant gene expression are observed in deacetylated PGC1α and FOXOa3. Therefore, antioxidant therapy aims to reduce the oxidative damage of Nrf2 and SIRT1 activation by polyphenolic substances [180,181,182,183]. Some of them were recently demonstrated to be involved in Nrf2 modulation as well as interfering with inflammation- and oxidation-related pathways and players in human diseases (Table 2).

Furthermore, research has demonstrated that SIRT1 may enhance OS by upregulating Nrf2 protein levels [190].

KEAP1 and PTEN suppress the transcriptional activity of Nrf2 under normal homeostatic settings. By directly interacting with Nrf2, KEAP1 facilitates the protein’s ubiquitination and eventual proteasomal breakdown. Moreover, PTEN removes 3-phosphoinositide (PIP3) necessary for AKT activation, which activates GSK3β and phosphorylates Nrf2. Antioxidants that function in vivo as electrophiles alter and inhibit PTEN and KEAP1. The relationship between KEAP1 and Nrf2 is broken and the half-life of Nrf2 is extended when KEAP1 is oxidized. Furthermore, electrophiles block the redox-sensitive phosphatase PTEN, which permits the accumulation of PIP3, the activation of AKT, and the inactivation of GSK3β. The production of antioxidant defense genes is induced by Nrf2, which is translocated to the nucleus and attaches onto the electrophile response element (EpRE) under these circumstances. Furthermore, by activating the transcription factors FOXOa3 and PGC1α, respectively, antioxidant substances alter the SIRT1 pathway in favor of MB and the antioxidant defense response [191].

### The Crosstalk Between SIRT1 and KEAP1/Nrf2/ARE Anti-Oxidative Pathway

The controlling role of the Sirt1-KEAP1/Nrf2/ARE signaling pathway in numerous physiological progressions has provoked many scientists [192]. SIRT 1 increases Nrf2 expression and promotes Nrf2 to the ARE region, consequently triggering ARE in the cells, acting as the upstream of the Nrf2/ARE pathway. The downregulation of the Sirt1/PGC-1α/Nrf2 pathway causes exposure of wild-type p53 cancer cells to OS [193]. The anticancer effects of Nrf2 were revealed in 2006, once hyperactivation of Nrf2 was found in lung cancer cell lines [194]. After this finding, Nrf2 overactivity was detected to occur in other cancer types as well. Multiple mechanisms have been shown to lead to Nrf2 overactivation. These include mutations in NFE2L2 and KEAP1 genes, epigenetic mechanisms, and various proteins that interrupt the binding between KEAP1 and Nrf2 [195].

The crosstalk between Nrf2 and its main controller KEAP1 is recognized to preserve redox, metabolic, and protein homeostasis, as well as to regulate inflammation and mitochondrial dysfunction. Under the normal physiological circumstances, KEAP1 binds to Nrf2 in the cytoplasm and inhibits Nrf2 transcriptional action via proteasomal degradation of Nrf2 protein [196]. The Nrf2–KEAP1 axis was revealed as vital against illnesses associated with OS and inflammation. However, recent research shows that Nrf2 signaling can coordinate cell-intrinsic defensive activities as well as classical antioxidant, detoxification, and homeostatic functions [197].

There is significant indication that SIRT1 is an essential controller of energy metabolism as well as of many vital functions [198]. SIRT1 regulates MB through PGC-1α and the oxidation of energy metabolic substrates. Furthermore, NAD+ is a strong stimulator of SIRT1, which makes SIRT1 a radar of metabolic homeostasis. Dysregulation of SIRT1 is related to various age-associated disorders such as cardiovascular and neurodegenerative diseases, and cancer [199]. SIRT1 could block some transcription factors, which are working in the control of cellular redox balance. For example, SIRT1 stops the transactivation ability of Nrf2 by deacetylating the Lys588 and Lys591 residues, which afterward inhibits the binding of Nrf2 to ARE. It was also observed that SIRT1-facilitated deacetylation of the Nrf2 protein terminated the transcription of antioxidant genes and subsequently, Nrf2 was moved out of the nuclei into the cytoplasm [200,201]. Research has shown that SIRT1 stimulates the action of Nrf2 and controls the expression of Nrf2 downstream genes, like SOD, and that the lack of SIRT1 results in the downregulation of Nrf2 expression and also enhances the stability of Nrf2 [202,203].

**Figure 6:**
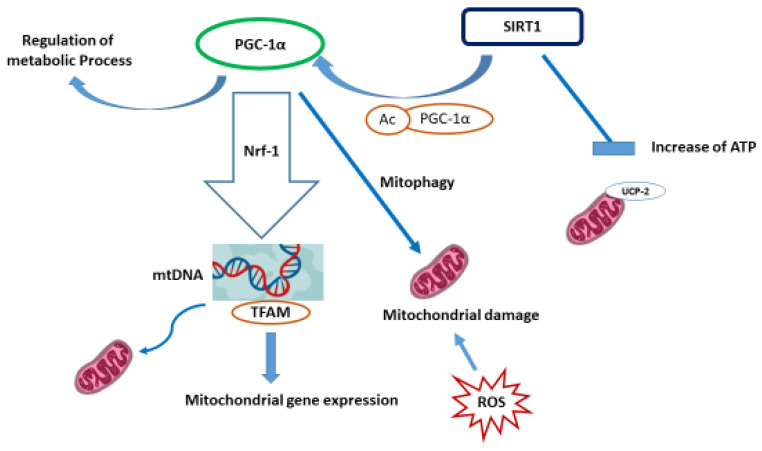
A schematic representation of SIRT1-mediated mitochondrial processes.

A schematic representation of SIRT1-mediated mitochondrial processes is presented in Figure 6. SIRT1 can interact with transcription factors or mitochondrial proteins to ensure healthy mitochondrial function. Furthermore, SIRT1 can increase ATP levels by suppressing Uncoupling Protein 2 (UCP-2) in the inner mitochondrial membrane. It can deacetylate PGC-1α, triggering its activation and increasing mitochondrial gene expression through Nrf-1 and TFAM, thus supporting mitochondrial biogenesis. PGC-1α may also participate in mitophagy, taking part in various metabolic processes.

## 6. Nrf2 and Mitochondrial Antioxidants

Maintaining healthy mitochondria requires proper balance of ROS homeostasis, calcium, mitophagy, and fission [204,205,206]. Since Nrf2 is a nuclear factor that senses ROS, imbalances in their quantity caused by overproduction by mitochondria, incapacity to eliminate them, or other causes, can affect the amount of Nrf2 in the cell as it tries to adapt, changing many cellular actions, such as glucose and lipid homeostasis [206]. As a result of the activation of TCA enzymes and respiratory chain components, a boost in MB, accompanied by a corresponding rise in OxPhos activity, causes an elevation in the generation of internally generated ROS via oxidative metabolism [142].

Human cells have an effective defense system to handle ROS with the help of both non-enzymatic and enzymatic antioxidants: catalase (exist primarily in peroxisomes and fewer in mitochondria), GR (exist in mitochondria), glutathione-S-transferase (exist in cytosol), and three forms of superoxide dismutases [207] (Mn-SOD, SOD1,SOD2, and SOD3) (Figure 6). OS, which occurs as a result of exposure to high concentrations of ROS in mitochondria, has been shown to be controlled by antioxidant molecules. For example, the mitochondrial antioxidant enzyme MnSOD has been shown to significantly worsen ischemic brain injury in its absence [208]. In another study, increased susceptibility to ICH was found in experimental animals with SOD1 deficiency. Increased expression of enzymes such as CAT and GPx in mitochondria was also observed to have increased neuroprotective effects against ischemic stroke and hemorrhagic stroke [209,210]. Studies have shown that the expression of enzymes involved in NADPH production, such as isocitrate dehydrogenase 1 (IDH-1), glucose-6-phosphate dehydrogenase (G6PD), malic enzyme 1 (ME-1), and 6-phosphogluconate dehydrogenase (PGD), can be controlled by Nrf2 [211]. Nrf2 can increase the activity of thioredoxins (TRXs) and peroxiredoxins (PRXs), which are used to scavenge oxidant particles in mitochondria. In addition, Nrf2 plays a crucial role in increasing the expression of GSH biosynthesis enzymes, which play a crucial role in the formation and maintenance of mitochondrial GSH stores [212].

Low molecular mass compounds possess antioxidant properties; these include flavonoids, carotenoids, GSH, ubiquinol, vitamins A, C, and E, and other antioxidants such as albumin.

**Figure 7:**
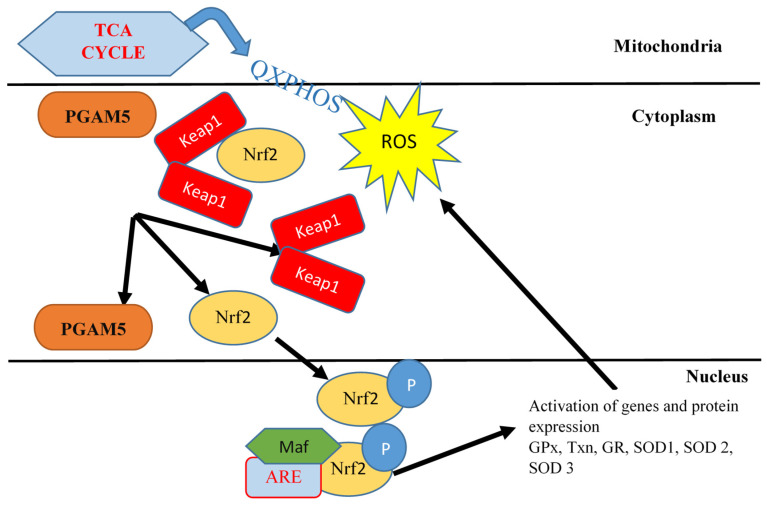
The stimulation of ARE-associated genes and antioxidant proteins, including thioredoxin (TXn), GR, GPx, SOD 1, SOD 2, and SOD 3, is caused by the modification of Nrf2 by mitochondrial ROS. Excess ROS are produced when there is a redox imbalance, which triggers the discharge of Nrf2 from KEAP1. This protein then moves to the cell nucleus and connects to ARE to initiate the transcription of antioxidant enzymes. A key player in mitochondrial activities, PGAM5 cooperates with Nrf2 and KEAP1 to form the Nrf2-KEAP1-PGAM5, which further emphasizes the critical part PGAM5 plays in the preservation of mitochondrial activities.

Recent studies have shown that the NRF2-PGAM5-KEAP1 complex plays an important role in cell health and disease prevention. It is also thought to play a role in determining mitochondrial functional mechanisms and cell death pathways [213]. Especially in neurodegenerative diseases, disruption or differentiation in the NRF2-PGAM5-KEAP1 structure may cause malfunction of mitochondrial processes necessary for neuronal resistance against OS [214] (Figure 7). One study observed that the NRF2-PGAM5-KEAP1 complex was disrupted during cardiotoxicity, resulting in decreased levels of PGAM5 and NRF2. This complex has been shown to regulate apoptosis, defend tumor cells against oxidative damage, and, when necessary, induce cell death in cases of pathological development [215]. Studies demonstrate the NRF2-PGAM5-KEAP1 complex’s ability to mediate cell defense and protect against disease. In cases of OS, it supports mitochondrial function and cellular homeostasis [216].

Conversely, lipophilic compounds, namely carotenoids, α-tocopherol, and ubiquinol are primarily found in cell membranes. It is possible that various antioxidant enzymes are connected [192]. Apart from its roles in MB as well as mitochondrial homeostasis, Nrf2 is also known to play an essential part in upholding cellular redox homeostasis by modifying the creation of ROS through the regulation of GSH, thioredoxin, and NADPH biosynthesis, utilization, and regeneration [71]. In response to oxidizing circumstances, Nrf2 activation increases a variety of mitochondrial antioxidant enzymes, including GR, GPx, thioredoxin 2, peroxiredoxin 3 (Prdx3), peroxiredoxin 5 (Prdx5), and SOD2 (Figure 7). However, the precise processes remain incompletely comprehended [172,217,218,219,220,221,222,223].

Nrf2 controls cytoprotective reactions against stress-induced ROS [224]. If the cells are not under OS, the Nrf2 protein amounts are small since proteasomes subsequently cause degradation. In the presence of OS or Nrf2-activating molecules, KEAP1 no longer has ubiquitin ligase activity due to oxidation of the cysteine residues. Then, Nrf2 is phosphorylated and enters into the cell nucleus. Finally, Nrf2 can attach to small proteins to make heterodimers. Heterodimers eventually bind to the ARE in the genes regulated by Nrf2 [225,226].

## 7. Conclusions

Studies on oxidative stress, which intensified in the 1970s, first led to the idea that many diseases and aging may be closely linked to free radicals and oxidant molecules [227]. Years of research in this field have proven that oxidative damage plays a significant role in the development of various pathological events. Therefore, it is known that preventing or controlling this damage will lead to significant improvements in disease treatment. A wide range of studies are being conducted to this end.

In one study, nanoenzymes that target mitochondria and are able to prevent oxidative damage against retinal neovascularization have been developed [228]. While oxidative damage is known to play a role in the development of many diseases, numerous studies are being conducted specifically on its active role in tumor development. Increased cell damage can induce apoptosis in cancer cells [229]. One study predicted that oxidative damage-induced tumor development would be suppressed by nanocomposites loaded with an MutT homolog 1 (MTH1) inhibitor [230]. An interesting review presents some important findings suggesting that the Nrf2/KEAP1 signaling pathway plays a significant role in the development of periodontitis-type inflammation. The effects of OS on decreased Nrf2 expression in the gingival tissues of periodontitis patients and the resulting pathologies are discussed using in vitro and in vivo models [231]. Current research demonstrates the protective and defensive roles of Nrf2 in the context of neurodegenerative diseases. Another review has detailed the potential role of Nrf2 in preventing neurodegenerative diseases and its ability to regulate antioxidant defense systems [232]. A review of non-alcoholic fatty liver disease (NAFLD) focused on treatment processes involving Nrf2. There is currently no approved drug molecule targeting Nrf2 for NAFLD treatment. However, this article focuses on the role of Nrf2 in NAFLD pathogenesis and presents some natural products that target Nrf2 or the Nrf2 pathway for NAFLD treatment [233]. Another review summarizes the relationships between the Keap1-Nrf2 signaling pathway and tumor development. The need for cancer treatment and some clinical applications utilizing this pathway is discussed. This review also addresses some of the dilemmas we encounter in this signaling pathway. From one perspective, Nrf2 activity can protect cells from oxidative and electrophilic stress, but in other cases, increasing Nrf2 activity can enhance cancer cell survival and proliferation. This dilemma is still under investigation and deserves significant attention in clinical practice. Therefore, investigating the mechanisms of this pathway at the molecular level will be a key target for the treatment of many pathological conditions, including other cancers and neurodegenerative diseases, and for drug development [234].

Although the associations between metabolic syndromes and mitochondrial modifications are not well understood, it is clear that OS in the cell may cause structural and functional modifications in the mitochondria. These changes activate cell signaling pathways and produce excess ROS which eventually lead to organ failure and diseases. Thus, antioxidant molecules that may control the generation of excessive ROS could be the possible beneficial answer to increasing mitochondrial condition in numerous diseases [235,236,237].

Screenings of possible Nrf2 inhibitors recognized an increasing number of natural and synthetic molecules preventing Nrf2 activation [238]. It has been thought that elevated amounts of mtROS can block the mechanisms that trigger Nrf2, causing the inhibition of Nrf2 action. Nevertheless, the diverse places of ROS production or alteration in reactive species generated could be interfering in Nrf2 inhibition [239]. The research continues and it is possible that Nrf2 activity will be considered as a target directly. This strategy may involve either activation or inhibition of Nrf2 activity. So far, only one compound as an Nrf2 activator (dimethyl fumarate) is approved for clinical use in MS patients [240].

Studies have reported that many natural and synthetic molecules have Nrf2 modulatory effects. However, all of these compounds have properties that make them unsafe for clinical use or have not yet been fully evaluated. Natural compounds generally present a picture of safety but weak efficacy. Most are nonspecific, limiting their clinical applications. Furthermore, most identified small molecule inhibitors have been obtained through high-throughput screening using the ARE-luciferase reporter gene system. The Nrf inhibition mechanisms, specificities, and toxicities of these molecules are still under investigation [241]. A significant problem encountered in studies of the Nrf2 protein is that its structure is not yet fully understood. While studies on Nrf2’s active site or allosteric pocket are ongoing, a complete mapping has not been achieved. Furthermore, Nrf2 exhibits a disordered profile when not bound to the KEAP1 protein, making it difficult to design new molecules that counteract it.

Therefore, more thorough research is needed to clarify the relevance of the relationship between Nrf2 and mitochondrial function. Since Nrf2 signaling acts as a “rheostat” in a number of diseases, additional translational research is required to verify that using this adaptable stress-induced cell response system is essential for restoring homeostasis and preserving human health. The precise molecular processes of Nrf2 inhibitors, as well as their safety and clinical suitability, require further research.

In conclusion, the development of Nrf2 modulator molecules against many diseases offers a promising strategy and market prospects. However, clinical studies in this area are not yet sufficient. Future studies in this area will lead to the development of potent and targeted molecules. However, effective compounds still have a long way to go before they can be used as clinical therapeutics.

## Figures and Tables

**Table 1:** Common Nrf2 activators and their ameliorating effects on mitochondrial dysfunctions that cause disorders.

Nrf2 Activator	Nature	Efficacy Endpoint	Reference
Sulforaphane (SFN)	Organosulfur compound	Major protective phytochemical against neurodegeneration	Kamal et al. (2020) [100]
1,4-diphenyl-1,2,3-triazole	1,4-disubstituted 1,2,3-triazole compound	Promote total mitochondrial homeostasis by motivating mitophagy	Kitaoka et al. (2019) [40]
P62-mediated mitophagy inducer (PMI)	Regulator compound of mitophagy	Activates mitochondrial autophagy	East et al. (2014) [92]
Bardoxylone	Oleanolic acid derivatives of semi-synthetic compound	Robust inducer of the Nrf2 pathway, leading to antioxidant and anti-inflammatory effects	Sun et al. (2021) [96]
RTA-408	Antioxidant inflammation modulator	Develop mitochondrial function	Sun et al. (2020) [94]
Dimethyl fumarate (DMF)	Methyl ester of fumaric acid compound	Activator of Nrf2	Majkutewicz (2022) [101]
TBE-31	Tricyclic compound	Induction of phase-2 cytoprotective pathways	Honda et al. (2011) [102]
tBHQ	Quinone compounds	Aryl hydrocarbon receptor ligand	Probst et al. (2015) [103]
Resveratrol	Natural phenol compound	Antioxidant and neuroprotective	Han et al. (2020) [104]
tHIQ	Tetrahydroisoquinoline compound	Cytoprotective oxidative stress response	Richardson et al. (2015) [105]

**Table 2:** Recent natural substances containing OH moieties and targeting Nrf2.

Name	Structure	Biological Activity
Methylnissolin (astrapterocarpan)	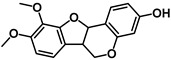	It modulates signaling pathways such as RIPK2/ASK1, PI3K/AKT, IκB/NF-κB, MAPK, and Nrf2/HO-1 in models of adaptive immunity and metabolic inflammation [184]
Hinokitiol (β-thujaplicin)	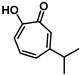	It significantly attenuates H_2_O_2_-induced cytotoxicity and ROS accumulation decreases MDA concentration [185]
Dioscin	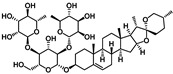	It increases the expression of LC3, Beclin-1, and Nrf2 and decreases P62 and KEAP1. Furthermore, it inhibits the levels of ROS and MDA, promoting SOD and CAT. It can also significantly downregulate the expression of TGF-β and Collagen I [186]
Luteolin	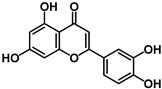	It modulates NF-κB, Nrf2, MAPK, JAK1/STAT3, PI3K/AKT, and pyroptosis, suppressing inflammatory mediators and oxidative stress [187]
Baicalein	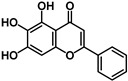	It modulates oxidative stress through Nrf2 activation and neuro-inflammation via NF-ĸB and MAPK inhibition, and mitochondrial protection [188]
Epigallocatechin gallate (EGCG)	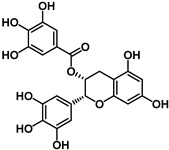	EGCG effectively promotes autophagy and targets Nrf2-ARE, NF-κB, and MAPK [189]

## Data Availability

No new data were created or analyzed in this study. Data sharing is not applicable to this article.
